# Isosorbide and nifedipine for Chagas' megaesophagus: A systematic review and meta-analysis

**DOI:** 10.1371/journal.pntd.0006836

**Published:** 2018-09-28

**Authors:** Celina Borges Migliavaca, Cinara Stein, Verônica Colpani, Sandro René Pinto de Sousa Miguel, Luciane Nascimento Cruz, Roberto Oliveira Dantas, Maicon Falavigna

**Affiliations:** 1 Institute for Education and Research, Hospital Moinhos de Vento, Porto Alegre, Brazil; 2 Federal University of Rio Grande do Sul, Porto Alegre, Brazil; 3 Centro Universitário FADERGS, Porto Alegre, Brazil; 4 Faculdade Meridional–IMED, Passo Fundo, Brazil; 5 National Institute of Science and Technology for Health Technology Assessment, Post Graduate Program in Epidemiology, Federal University of Rio Grande do Sul, Porto Alegre, Brazil; 6 Department of Medicine, Ribeirão Preto Medical School, University of São Paulo, Ribeirão Preto, São Paulo, Brazil; 7 Department of Health Research Methods, Evidence and Impact, McMaster University Health Sciences Centre, Hamilton, Canada; Baylor College of Medicine, UNITED STATES

## Abstract

**Background:**

Chagas disease is a neglected tropical disease. About 6 to 8 million people are chronically infected and 10% to 15% develop irreversible gastrointestinal disorders, including megaesophagus. Treatment focuses on improving symptoms, and isosorbide and nifedipine may be used for this purpose.

**Methodology:**

We conducted a systematic review to evaluate the effectiveness of pharmacological treatment for Chagas’ megaesophagus. We searched MEDLINE, Embase and LILACS databases up to January 2018. We included both observational studies and RCTs evaluating the effects of isosorbide or nifedipine in adult patients with Chagas’ megaesophagus. Two reviewers screened titles and abstracts, selected eligible studies and extracted data. We assessed the risk of bias using NIH ‘Quality Assessment Tool for Before-After (Pre-Post) Studies with No Control Group’ and RoB 2.0 tool. Overall quality of evidence was assessed using GRADE.

**Principal findings:**

We included eight studies (four crossover RCTs, four before-after studies). Three studies evaluated the effect of isosorbide on lower esophageal sphincter pressure (LESP), showing a significant reduction (mean difference −10.52mmHg, 95%CI −13.57 to−7.47, very low quality of evidence). Three studies reported the effect of isosorbide on esophageal emptying, showing a decrease in esophageal retention rates (mean difference −22.16%, 95%CI −29.94 to −14.38, low quality of evidence). In one study, patients on isosorbide reported improvement in the frequency and severity of dysphagia (moderate quality of evidence). Studies evaluating nifedipine observed a decrease in LESP but no effect on esophageal emptying (very low and low quality of evidence, respectively). Isosorbide had a higher incidence of headache as a side effect than nifedipine.

**Conclusions:**

Although limited, available evidence shows that both isosorbide and nifedipine are effective in reducing esophageal symptoms. Isosorbide appears to be more effective, and its use is supported by a larger number of studies; nifedipine, however, appears to have a better tolerability profile.

**Trial registration:**

PROSPERO CRD42017055143.

ClinicalTrials.gov CRD42017055143.

## Author summary

Chagas disease is a chronic neglected tropical disease that has increased in prevalence in the last decade. About 10% of chronically infected patients develop the digestive form of the disease. Megaesophagus is a common manifestation, and symptoms include difficulty or discomfort in swallowing and regurgitation. Treatment approaches include dietary interventions, medications, and endoscopic and surgical interventions. Regarding pharmacological treatment, only a few small studies have evaluated the effects of isosorbide and nifedipine, mainly on surrogate outcomes. According to our systematic review, the use of isosorbide reduced lower esophageal sphincter pressure, esophageal retention after meal ingestion, and the frequency and severity of dysphagia, while nifedipine reduced esophageal retention after meal ingestion. In light of this, both medications are effective in the treatment of symptoms associated with Chagas disease. Isosorbide appears to be more effective, and its use is supported by a larger number of studies; nifedipine, however, appears to have a better tolerability profile. Both drugs are valid alternatives, and the decisions about pharmacological treatment should be tailored to each patient.

## Introduction

Chagas disease, also known as American trypanosomiasis, is an infectious zoonosis caused by the protozoan parasite *Trypanosoma cruzi*. Once confined to Latin America, it has now spread to other continents. It is estimated that about 6 to 8 million people are infected worldwide. Infection is lifelong and can be life threatening, killing more than 10 000 people every year. Despite its relevance, it is considered by the World Health Organization a neglected tropical disease [1, 2].

Chagas disease is transmitted by triatomine vectors (popularly known as kissing bugs), vertically (from mother to fetus), orally (by ingestion), by blood transfusion, by organ transplants, and by laboratory accidents. During the first weeks or few months, the disease presents in its acute form, which has no or only mild symptoms, such as fever, fatigue, and headache; thus, infection often goes unnoticed. The host’s immune system then controls parasite replication, and patients enter the chronic phase. Most patients remain asymptomatic, but approximately 30% of infected people develop medical complications from Chagas disease over the course of their lives, usually several years or even decades after the initial infection. The disease mainly affects the heart, digestive system, and nervous system [3, 4].

Digestive disorders are the second most common manifestation of Chagas disease, affecting about 10% to 15% of patients [5]. There are geographical variations in the prevalence and severity of this condition, which may be associate with the distribution of different *T*. *cruzi* strains. They are more frequent in central and southern South America (including Argentina, Bolivia, Brazil, Chile, Paraguay and Uruguay) and very rare in other regions [6–8]. Gastrointestinal symptoms are the result of an irreversible enteric nervous system impairment caused by the parasite *T*. *cruzi*. Any organ of the digestive system can be affected, but the esophagus and colon are most often injured, causing megaesophagus and megacolon [6]. The esophagus is usually the first affected organ and megaesophagus is the most prevalent manifestation of gastrointestinal Chagas disease [6, 9]. The most common symptoms of megaesophagus are dysphagia, odynophagia, and esophageal regurgitation. Even though these symptoms do not usually lead to death, they are associated with an increased risk of cancer and may impact quality of life [4, 6, 10]. The diagnosis of Chagas’ megaesophagus is based mainly on clinical history, symptoms, barium esophagogram and manometry. Further details about clinical aspects of Chagas’ megaesophagus are presented in Table 1.

**Table 1:** Clinical characteristics of Chagas’ megaesophagus.

Prevalence [2, 5, 11]	Chronic Chagas disease: 6 to 8 million patients worldwide. Digestive form: 10 to 15% of chronic patients. In endemic regions, about 7 to 10% of patients with Chagas disease present radiologic evidence of esophageal disorders and 3% present dilated esophagus.
Pathophysiology [12]	Denervation of the enteric nervous system, caused by the presence of the parasite in the tissue and immunological response of the host. It affects both parasympathetic and sympathetic neurons from the submucosal and myenteric plexuses.
Clinical manifestations [11]	Main symptoms are dysphagia, odynophagia, regurgitation, retrosternal pain and malnutrition.
Diagnosis [13]	Diagnosis is based on clinical assessment of signals and symptoms. Complementary diagnostic tests often used for are esophageal manometry, barium esophagogram, chest X-ray and endoscopy.
Staging [14]	Most used staging system for Chagas esophageal disease is the Rezende’s classification: I: no esophageal dilatation, with minimal retention; II: moderate esophageal dilatation, with some retention and uncoordinated motor activity; III: large esophageal dilatation, with great retention and weak or absent motor activity; IV: severe esophageal dilatation, atonic and elongated esophagus.
Etiological treatment [13]	Benznidazole and nifurtimox does not seem to have an effect on the progression of digestive manifestations. These medications may be used to prevent other complications of chronic Chagas disease, such as cardiovascular manifestations.
Symptomatic treatment [5, 13]	• Nutritional modifications: eat in small portions, eat with liquids, avoid very hot, cold and seasoned foods • Pharmacological treatment: isosorbide dinitrate and nifedipine • Others: botulinum toxin injection, pneumatic balloon dilatation, surgery (cardiomyotomy) Patients with stage I and II may have a good response with nutritional modifications and pharmacological treatments. Patients with stage III and IV, and some with stage I and II non-responsive to diet modification and/or drugs, usually require additional intervention, including surgery.

The changes in esophageal function caused by *T*. *cruzi* are similar, but not equal, to those caused by idiopathic (primary) achalasia. Although differences have been described, both conditions are treated similarly [15, 16]. Treatment always includes dietary adjustments, but they may not be sufficient for some patients. In these cases, pharmacological, endoscopic and surgical interventions can be recommended with the main purpose of decreasing lower esophageal sphincter pressure (LESP), improving esophageal emptying, and relieving symptoms of dysphagia [17, 18] (Table 1).

Regarding pharmacological treatment, the most commonly used medications are isosorbide dinitrate and nifedipine [6]. Isosorbide dinitrate (2.5-5mg sublingually, 15 minutes before meals) releases nitric oxide, which activates the enzyme guanylate cyclase, leading to smooth muscle relaxation [13, 19]. Nifedipine (10mg sublingually, 30 minutes before meals) is a calcium channel blocker that prevents calcium-dependent myocyte contraction, also leading to muscle relaxation [13, 19]. These drugs appear to be effective in relieving symptoms, but their use is controversial because of the high incidence of side effects and no change in the course of the disease [6, 18]. Only a few small studies have evaluated the use of these medications in patients with Chagas disease, and there is no systematic review on this topic. Therefore, the objective of this systematic review was to evaluate the effectiveness of isosorbide and nifedipine *versus* no treatment for esophageal manifestations of Chagas disease in adult patients and to determine the frequency of side effects.

## Methods

### Protocol and registration

This systematic review is reported according to the PRISMA Statement [20] (S1 Appendix) and was conducted following the recommendations of the Cochrane Handbook for Systematic Reviews of Interventions [21]. The study protocol was registered with the International Prospective Register of Systematic Reviews (PROSPERO), under the registration number CRD42017055143 (S2 Appendix).

### Search strategy

We searched MEDLINE (via PubMed), Embase and LILACS databases to retrieve potentially relevant articles from inception to January 2018. We also screened the reference lists of identified publications for additional studies and contacted authors for further information as needed. We conducted two independent searches, one for each intervention (isosorbide and nifedipine). Search terms included “Chagas disease”, “*Trypanosoma cruzi”*, “isosorbide”, and “nifedipine”. Keywords related to outcomes of interest and publication type were not included to enhance the sensitivity of the search. No language or publication date restrictions were imposed. Search terms were tailored to each database, and the complete search strategies are shown in S3 Appendix.

### Eligibility criteria and outcomes of interest

We included studies that met the following criteria: (A) observational studies or clinical trials; (B) studies that assessed the effects of isosorbide or nifedipine on esophageal symptoms or esophageal function in patients with Chagas disease; and (C) individuals aged >18 years with digestive or cardiodigestive form of Chagas disease, according to original studies definitions. We excluded reviews, letters, and editorials.

The outcomes of interest were (A) esophageal symptoms (e.g. dysphagia and regurgitation), (B) esophageal function (e.g. LESP and esophageal emptying), and (C) adverse events. We did not include other gastrointestinal symptoms caused by Chagas disease, because isosorbide and nifedipine are used only for esophageal symptoms.

### Study selection and data extraction

In order to screen and select eligible studies, we combined the results from both searches. All identified citations were entered into a software for reference management, and duplicates were excluded. Two independent reviewers (CBM and CS) screened the titles and abstracts of all potentially relevant articles identified by the searches, and studies not meeting the eligibility criteria were excluded. The same reviewers assessed the full-text articles of selected abstracts for inclusion according to the pre-specified eligibility criteria. If the study was reported in duplicate, the study published earlier or the one that provided more information was included. Independently, the same reviewers extracted data from the full text of included studies using a pre-designed data extraction form. Data extracted included study characteristics and outcomes of interest. When needed, data were extracted from figures or graphs using WebPlotDigitizer [22]. Disagreements regarding study eligibility or data extraction were discussed between the two reviewers. If consensus was not reached, a third reviewer (VC) arbitrated.

### Risk of bias assessment

Two reviewers (CBM and CS) independently assessed the methodological quality of included studies. We used the NIH ‘Quality Assessment Tool for Before-After (Pre-Post) Studies with No Control Group’ [23] for all observational before-after studies and the RoB 2.0 tool [24] for all crossover clinical trials. Disagreements regarding the methodological quality of the studies were discussed between the two reviewers. If consensus was not reached, a third reviewer (VC) arbitrated.

The overall quality of evidence was assessed using GRADE [25].

### Data analysis

Where possible, data were pooled using a meta-analytic approach. A random-effects model, with DerSimonian and Laird’s variance estimator, was used, and the results were presented as mean difference or pooled prevalence, with 95% confidence intervals (95%CI). A *P* value ≤ 0.05 was considered statistically significant. Statistical heterogeneity among studies was assessed using Cochran’s Q test and the I^2^ statistic. All meta-analyses were performed using the R statistical software version 3.3.3, with meta package version 4.8–1 [26, 27].

When a study did not report the standard deviation (SD), one of the following three strategies was used: estimation of individual patient data from the study’s graphs and calculation of mean and SD; calculation of SD from *P* value; or input of the highest SD found in other studies for the same outcome.

Studies not included in the meta-analysis were presented descriptively.

## Results

### Description of studies

**Fig 1:**
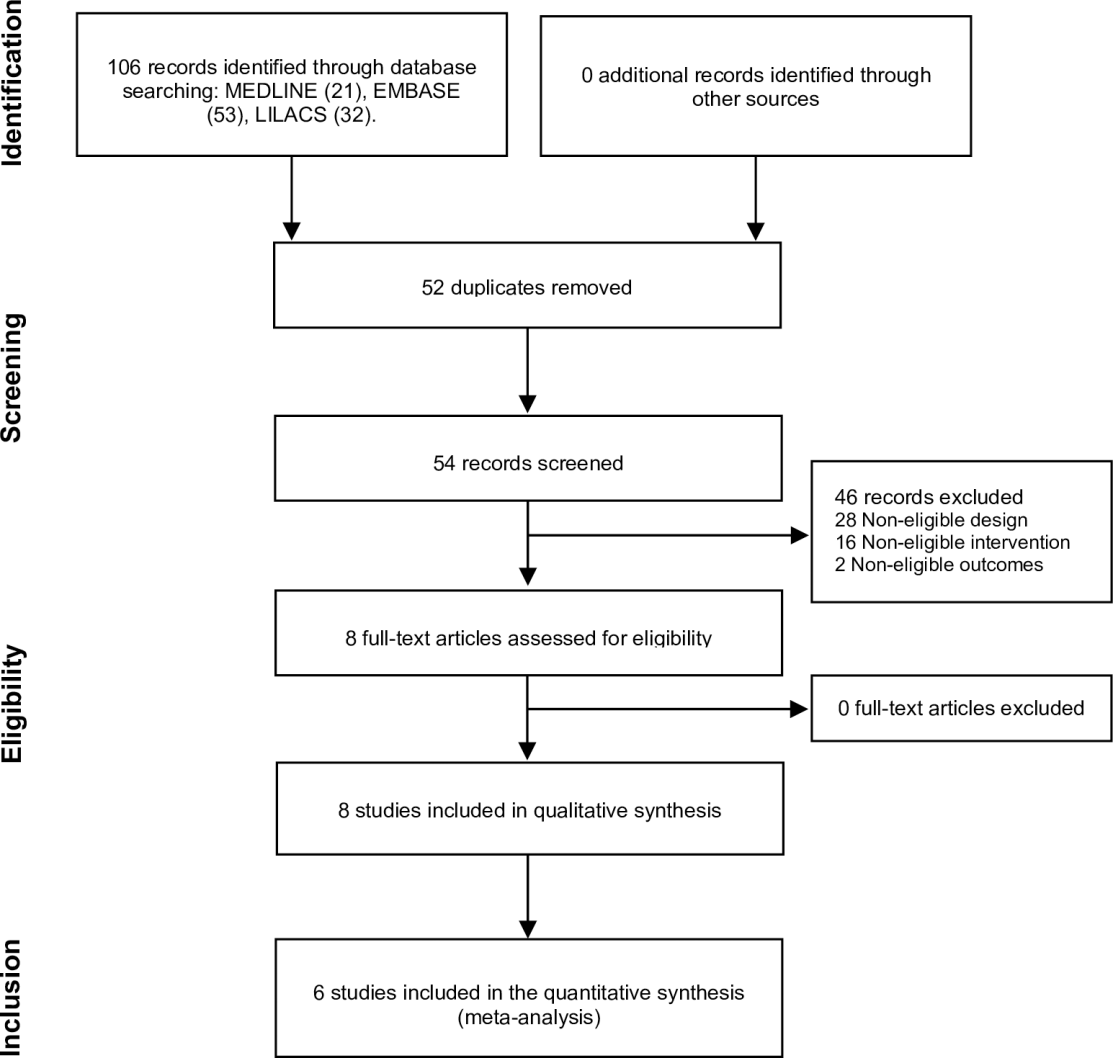
Flowchart of study selection. Six studies evaluated isosorbide, one evaluated nifedipine, and one evaluated both. Four studies had a before-after design, and four were crossover trials.

A total of 66 studies were retrieved for ‘isosorbide’ and 40 for ‘nifedipine’. Of these 106 studies, eight met the eligibility criteria and were included in our review. Fig 1 shows the flow diagram of study selection, and Table 2 shows the main characteristics of included studies.

**Table 2:** Characteristics of studies included in the systematic review.

Author, Year, Country	Population	Rezende’s classification	N	Median age (range), yrs	Male	Intervention	Outcomes
**Before and after studies**							
Dantas et al., 1987, Brazil [28]	Chagas’ patients with dysphagia for more than 1 year and esophageal disease confirmed by radiology and manometry	I: 12; II: 13; III: 3	28	49 (27–66)	50%	Isosorbide dinitrate, 5mg, sublingually	LESP (measured by manometric method); Side effects
Dantas et al., 1988, Brazil [29]	Chagas’ patients with dysphagia for more than 1 year and esophageal disease confirmed by radiology and manometry	I:17; II: 7	24	50* (35–62)	45%	Isosorbide dinitrate, 5mg, sublingually	LESP (measured by manometric method)
Matsuda et al., 1995, Brazil [30]	G1: Chagas’ patients with normal esophageal manometry; G2: Chagas’ patients with dysphagia and esophageal dysmotility	NR	G1: 15; G2: 9	G1: 54 (33–75); G2: 50 (33–72)	G1: 46%; G2: 33%	Isosorbide dinitrate, 5mg, sublingually	LESP (measured by manometric method)
Dantas et al., 1986, Brazil [31]	Chagas’ patients with dysphagia for more than 1 year and esophageal disease confirmed by radiology and manometry	I: 11; II: 4	15	49 (30–58)	46%	Nifedipine, 10mg, sublingually	LESP (measured by manometric method); Side effects
**Crossover RCTs**							
de Oliveira et al., 1994, Brazil [32]	Chagas’ patients with dysphagia and some with regurgitation, with esophageal disease confirmed by barium esophagogram	II: 6; III: 10; IV: 2	18	50 (23–66)	61%	Control (no medication); Isosorbide dinitrate, 5mg, sublingually; Cardiomyotomy	Esophageal emptying (assessed through retention of 11.1 MBq ^99m^Tc-phytate 5 minutes after meal ingestion, at three esophageal emptying scintigraphic studies performed on different days); Side effects
Ferreira-Filho et al., 1991, Brazil [33]	Chagas’ patients with dysphagia and esophageal disease confirmed by radiology	NR	23	57 (18–73)	35%	7 days of isosorbide dinitrate (5mg, sublingually) preceded or followed by 7 days of placebo	Frequency and severity of dysphagia and side effects, evaluated at regular interviews
Figueiredo et al., 1992, Brazil [34]	Chagas’ patients with dysphagia and some with regurgitation, with esophageal disease confirmed by barium esophagogram	NR	11	53 (28–75)	64%	Control (no medication); Isosorbide dinitrate, 5mg, sublingually; Nifedipine, 20mg, sublingually	Esophageal emptying (assessed through retention of 11.1 MBq ^99m^Tc sulphur colloid 5 minutes after meal ingestion, at three esophageal emptying scintigraphic studies performed on different days); Side effects
Rezende Filho et al., 1990, Brazil [35]	Chagas’ patients with dysphagia for more than 1 year and esophageal disease confirmed by radiology	II: 9; III: 7; IV: 2	18	42 (21–78)	61%	Control (no medication); Isosorbide dinitrate, 5mg, sublingually	Esophageal emptying (assessed through retention of ^99m^Tc-pertechnetate 5 minutes after meal ingestion, at two esophageal emptying scintigraphic studies performed on different days)

RCT: randomized clinical trial; LESP: lower esophageal sphincter pressure; NR: not reported; * Mean

### Risk of bias

The quality of all before-after studies was rated as fair [28–31]. All of them presented issues concerning lack of information about eligibility criteria, sample size calculation, blinding, and loss to follow-up. However, all clearly stated the objectives, interventions, and outcomes, statistically analyzed the results and presented *P* values for the analysis. Among crossover trials, only one study was rated as having a low risk of bias, with no major concerns [33]. The other three studies were rated as having a high risk of bias due to concerns related to randomization, allocation concealment, and blinding of patients and assessors [32,34,35]. Risk of bias assessment of included studies is summarized in S4 Appendix. Publication bias was not assessed due to the small number of studies.

### Effects of isosorbide

#### Lower esophageal sphincter pressure (LESP)

Three before-after studies evaluated the effect of isosorbide on LESP and were included in the meta-analysis [28, 29, 30]. In all studies, the authors measured LESP using manometric methods before and after isosorbide (5mg) sublingual administration. The complete dataset is shown in S5 Appendix.

**Fig 2:**
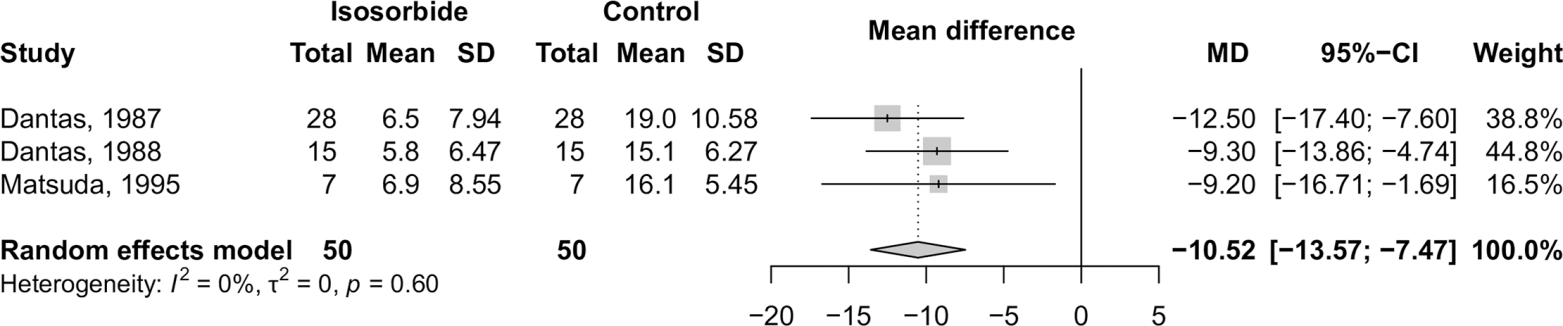
Effect of isosorbide on lower esophageal sphincter pressure 10 minutes after administration.

After 10 minutes of administration, isosorbide decreased LESP by 10.52mmHg (95%CI −13.57 to −7.47; p<0.0001), leading to improvement in dysphagia (Fig 2). These results were consistent for other time periods evaluated by the original studies (range: 5 to 60 minutes). Heterogeneity among studies was not detected (I^2^ = 0%), and the quality of evidence was rated as very low due to the high risk of bias and imprecision. The full GRADE assessment is available in S6 Appendix.

#### Esophageal emptying

Three crossover trials assessing the effect of isosorbide on esophageal emptying were included in the meta-analysis [32,34,35]. Immediately after isosorbide (5mg) sublingual administration or under basal conditions, the patients ingested a radiolabeled meal, and at the completion of the meal, imaging started using a scintigraphic technique. The percentage of radioactivity retained on the esophagus at 5 minutes after meal ingestion (on isosorbide and under basal conditions) was used to evaluate the effect of isosorbide on esophageal emptying. The complete dataset is shown in S5 Appendix

**Fig 3:**
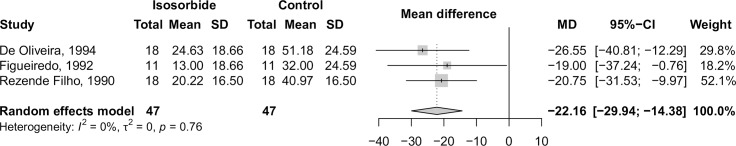
Effect of isosorbide on esophageal retention 5 minutes after meal ingestion. Esophageal retention is an indicator of esophageal emptying.

Isosorbide decreased esophageal retention by 22.16% (95%CI −29.94 to −14.38; p<0.0001), which is indicative of an increase in esophageal emptying (Fig 3). These results were consistent for other time periods evaluated by the original studies (range: 5 to 40 minutes). Heterogeneity among studies was not detected (I^2^ = 0%). The quality of evidence was rated as low due to the high risk of bias and imprecision (S6 Appendix).

#### Dysphagia and global well-being

Ferreira-Filho et al. (1991) [33] assessed the effect of isosorbide *versus* placebo on the frequency and severity of dysphagia and global well-being in 23 patients with Chagas disease. Patients took both isosorbide and placebo, for 1 week each, in a randomly allocated order. During the weekly interviews, patients were asked to rate the frequency and severity of dysphagia and global well-being compared with prior treatment. Scores for the frequency and severity of dysphagia were significantly lower after isosorbide treatment compared with prior treatment and placebo. Regarding global well-being, 12 out of 20 patients reported improvements compared with the pretreatment condition. However, there was no difference in scores between isosorbide and placebo, probably because of side effects. Three patients did not complete the study, one because of headache and fainting during isosorbide treatment. The quality of evidence was rated as moderate due to imprecision (S6 Appendix).

#### Side effects

Four studies evaluated side effects caused by isosorbide and were included in a meta-analysis of prevalence data, shown in S7 Appendix [28,32–34]. All studies reported cases of headache, and two of them assessed cases of palpitation and faintness.

The prevalence of headache after sublingual intake of isosorbide was 31.60% (four studies, 80 patients, 95%CI 11.65 to 61.82). Furthermore, 9.50% of patients (two studies, 39 patients, 95%CI 0.54 to 66.90) reported palpitation and 7.74% (two studies, 39 patients, 95%CI 2.52 to 21.41) reported faintness after isosorbide ingestion (S7 Appendix).

### Effects of nifedipine

#### Lower esophageal sphincter pressure (LESP)

Only one before-after study evaluated the effect of nifedipine on LESP in patients with Chagas disease [31]. Dantas et al. (1986) reported a decrease in LESP 35 minutes after nifedipine administration, and the effect lasted for about 20 minutes. However, this effect was not present in all patients: four out of 15 patients with Chagas disease showed no changes in LESP. The quality of evidence was rated as very low due to the high risk of bias and imprecision (S6 Appendix).

#### Esophageal emptying

Figueiredo et al. (1992) [34] assessed the effect of nifedipine on esophageal emptying in 11 patients with Chagas disease using a scintigraphic technique. There was no difference between food retention under basal conditions and after nifedipine administration. The quality of evidence was rated as low due to the high level of imprecision (S6 Appendix).

#### Side effects

Two studies reported side effects caused by nifedipine administration [31, 34]. The results were included in a meta-analysis of prevalence data, shown in S7 Appendix.

Nifedipine caused headache in 10.48% of patients (two studies, 26 patients, 95%CI 3.04 to 30.41) (S7 Appendix). No study reported cases of faintness or palpitation after nifedipine administration.

## Discussion

This review evaluated the effect of isosorbide and nifedipine on esophageal symptoms in patients with Chagas disease. Studies investigating the effect of isosorbide showed that this medication decreased LESP and esophageal retention; moreover, it improved the severity and frequency of dysphagia as reported by patients. Among the studies, mean baseline LESP was 17.4mmHg. The reduction of 10.52mmHg correspond to 1.33 standard deviation, which is considered a large magnitude of effect, indicating the intervention is highly effective in reducing LESP [36]. However, about 30% of patients had headache, and up to 10% reported faintness and palpitation. Studies evaluating nifedipine showed a reduction in LESP but no effect on esophageal retention. The most common side effect of nifedipine was headache, reported by about 10% of patients.

Even though digestive symptoms are a well-known manifestation of Chagas disease, recommendations for their treatment are often neglected. In a review of published guidelines for the management of Chagas disease, 10 documents were found, but only two of them provided recommendations for the pharmacological treatment of megaesophagus [37].

Both isosorbide and nifedipine led to improvement in esophageal symptoms. However, only one study, involving 11 patients, directly compared the two drugs of interest [34]. By indirectly comparing the effects of these medications on esophageal function, based on the results of our meta-analysis, isosorbide was superior compared to nifedipine, showing faster onset and longer duration of effects. Moreover, the body of evidence was more consistent for isosorbide than for nifedipine, with seven studies (146 patients) on isosorbide and only two studies (26 patients) on nifedipine.

Although current evidence potentially indicates greater certainty that isosorbide is more effective, it is important to consider potential side effects when choosing treatment. In our systematic review, from 30 to 50% of patients on isosorbide reported side effects such as headache, faintness, and palpitation, which may impact long-term adherence to the medication. The rate of side effects of nifedipine was lower, a result similar to that reported in studies of patients without Chagas disease [38, 39]. Although nifedipine is usually better tolerated, it should be avoided in patients with severe cardiomyopathy, a common manifestation of Chagas disease, due to the risk of hypotension and hydrosaline retention.

To our knowledge, this is the first systematic review to evaluate the effects of isosorbide and nifedipine in patients with Chagas disease. We performed a comprehensive literature search without language or date restrictions and systematically evaluated the risk of bias and quality of evidence for the proposed interventions. Included studies applied diagnostic and staging methods currently in use, enhancing the external validity of our findings. Additionally, we performed a meta-analysis of several outcomes of interest, thereby increasing statistical power and precision and making it possible to assess the consistency of the findings.

Our systematic review has some limitations, mostly due to the characteristics of included studies. First, the number of included studies is small, a consequence of the sparse number of publications in the field. Besides, all studies were conducted in Brazil, most of them in the same city. This may limit generalizability of the findings, which may be an issue of concern especially nowadays when Chagas disease has spread globally. Moreover, the studies were conducted at least 23 years ago, and since then the healthcare provided to patients has probably changed. Furthermore, all included studies were very small and had methodological limitations. Regarding outcome evaluation, most studies evaluated only surrogate outcomes for symptom improvement, and these measurements may not directly reflect clinical improvement. Besides, no study assessed the long-term effects of these medications; thus, our conclusions provide direct evidence only to short-term outcomes, and the long-term effects for these interventions are still unknown. It is important to note that our systematic review evaluated only studies of patients with Chagas disease; for the assessment of side effects, we did not include information from further studies conducted in other relevant fields, such as cardiology. Although including the results of patients without Chagas disease would increase the heterogeneity and indirectness of our findings, it could give more precision to the prevalence estimates of some side effects that may be similar in patients with and without Chagas disease.

Based on the findings of our systematic review, while also acknowledging the lack of rigorous studies such as long-term clinical trials, isosorbide and nifedipine are effective in the treatment of esophageal manifestations of Chagas disease. Isosorbide appears to be more effective, and its use is supported by a larger number of studies. However, nifedipine appears to have a better tolerability profile. Both drugs seem to be valid alternatives, and the decisions about pharmacological treatment should be tailored to each patient.

## Supporting information

S1 Appendix PRISMA checklist and flow diagram. (PDF)

S2 Appendix PROSPERO protocol. (PDF)

S3 Appendix Search strategy. (PDF)

S4 Appendix Risk of bias assessment of included studies. (PDF)

S5 Appendix Complete dataset for the effects of isosorbide and nifedipine on lower esophageal sphincter pressure (LESP) and esophageal emptying. (PDF)

S6 Appendix GRADE assessment. (PDF)

S7 Appendix Prevalence of side effects of isosorbide and nifedipine. (PDF)
