# Supplementary material for: Isosorbide and nifedipine for Chagas' megaesophagus: A systematic review and meta-analysis
Source: PLoS Negl Trop Dis. 2018 Sep 28;12(9):e0006836. doi: 10.1371/journal.pntd.0006836 (PMC6179300; doi:10.1371/journal.pntd.0006836)
Supplement: S2 Appendix — (PDF) [file pntd.0006836.s002.pdf]

National Institute for Health Research

Isosorbide and nifedipine for treatment of achalasia in patients with digestive clinical form of chronic Chagas disease: a systematic review

Cinara Stein, Celina Borges Migliavaca, Verônica Colpani, Luciane Nascimento Cruz, Maicon Falavigna

Citation

Cinara Stein, Celina Borges Migliavaca, Verônica Colpani, Luciane Nascimento Cruz, Maicon Falavigna. Isosorbide and nifedipine for treatment of achalasia in patients with digestive clinical form of chronic Chagas disease: a systematic review . PROSPERO 2017 CRD42017055143 Available from: http://www.crd.york.ac.uk/PROSPERO/display_record.php?ID=CRD42017055143

Review question

To evaluate the effect of isosorbide in gastrointestinal symptoms of chagasic patients.
To evaluate the effect of nifedipine in gastrointestinal symptoms of chagasic patients.

Searches

We will search MEDLINE (accessed by PubMed), EMBASE and LILACS databases to retrieve potentially relevant articles. Moreover, we will search on the website ‘clinicaltrials.gov’ and scan the reference lists of identified publications for additional studies.
Search terms will include relevant headings and keywords in the title, abstract and text, including terms as “Chagas disease”, “Trypanosoma cruzi”, “Isosorbide” and “Nifedipine”.
Keywords related to outcomes of interest and publication type will not be included to enhance sensitivity of search.

Types of study to be included

Observational investigations (cohort studies or case series), clinical trials and randomized clinical trials will be included.We will exclude experimental studies, case-control studies, cross-sectional studies, systematic reviews and meta-analyses, letters, and editorials.

Condition or domain being studied

Chagas disease, also known as American trypanosomiasis, is a zoonosis that affects about 8–12 million people in Latin America (1). It is estimated that some 40 million people are at risk of acquiring the infection (2).
Approximately 30% of infected people develop medical problems from Chagas disease over the course of their lives, usually several years or even decades after the initial infection. Chagas disease mainly affects the heart, nervous system and digestive system (3). Gastrointestinal manifestations are the second most common cause of complications due to Chagas disease. Approximately one third of patients can develop gastrointestinal motor disorders, such as achalasia of the cardia, disturbances of gastric emptying, altered intestinal transit and colon and gallbladder motor disorders (4,5). There are no specific treatments capable of restoring esophageal function, though partial recovery of esophageal peristalsis can be observed following clinical, endoscopic or surgical management (6).
The treatment with isosorbide and nifedipine induce sphincter pressure relaxation and can be used in some cases. However, these treatments are not always effective and side effects are frequent (7). In patients with Chagasic achalasia, isosorbide dinitrate has shown to reduce lower esophageal sphincter pressure (8) improve esophageal emptying (9) and to relieve dysphagia (6). However, the beneficial effect of isosorbide dinitrate for patients with Chagasic achalasia was often impaired by headache as a common side effect (10). Sublingual nifedipine, calcium channel blocker, also decreases lower esophageal sphincter pressure in patients with Chagasic achalasia (11). The use of these therapies is doubtful, since it is considered a palliative treatment and followed by side effects. Based on this, it is necessary to systematically review the effects of isosorbide and nifedipine in gastrointestinal symptoms of chagasic patients.
1.Pan American Health Organization, WHO Department of Control of Neglected Tropical Diseases.

Estimaci´on cuantitativa de la enfermedad de Chagas en las Americas. Montevideo, Uruguay 2006. OPS/HDM/CD/22506.
2. Schofield C, Jannin J, Salvatella R. The future of Chagas disease control. Trends Parasitol. 2006; 22:583–8.
3. Bern C., et al. Evaluation and treatment of Chagas disease in the United States: A systematic review. JAMA. 2007; 298:2171-81
4. Dantas RO, Godoy RA. The lower esophageal sphincter in patients with Chagas disease with peristalsis and aperistalsis. Arq Gastroenterol. 1983; 20:13-6.
5. Oliveira RB, Troncon LE, Dantas RO, Menghelli UG. Gastrointestinal manifestations of Chagas disease. Am J Gastroenterol. 1998; 93:884-9.
6. Pinazzo MJ, et al. Diagnosis, management and treatment of chronic Chagas' gastrointestinal disease in areas where Trypanosoma cruzi infection is not endemic. Gastroenterol Hepatol. 2010 Mar;33(3):191-200
7. Oliveira RB, Rezende Filho J, Dantas RO, Iazigi N. The spectrum of oesophageal motor disorders in Chagas disease. Am J Gastroentero. 1995; 90:1119–24.
8.Dantas R 0, Godoy R A, Oliveira R B, et al. Effect of isosorbide dinitrate and atropine on the lower esophageal sphincter pressure in chagasic patients. Acta Physiol Pharmacol Latinoam 1988; 38: 151-8.
9. Rezende Filho J, Oliveira R B, Dantas R 0, et al. Efeito do dinitrato de isossorbitol sobre o esvaziamento esofagiano no megaesbfago chagdsico. SZo Paulo Arq Gastroenterol 1990; 27: 115-9.
10. Ferreira Filho L P, Patto R J, Troncon L E A, et al. Use of isosorbide dinitrate for the symptomatic treatment of patients with Chagas' disease achalasia. A double-blind, crossover trial. Brazilian J Med Biol Res 1991; 24: 1093-98.
11. Dantas R 0, Godoy R A, Villanova M G, et a1. Effect of nifedipine on the lower esophageal sphincter pressure in chagasic patients. Brazilian J Med Biol Res 1986; 19: 205-9.

Participants/population
Adults (> 18 years old) affected by gastrointestinal Chagas disease.

Intervention(s), exposure(s)
We will include studies regarding the use of isosorbide or nifedipine to treat gastrointestinal symptoms in patients affected by gastrointestinal Chagas disease.

Comparator(s)/control
The comparator groups will be placebo group, standard care, no intervention, or different treatment drugs.

Primary outcome(s)
The primary outcome will be improvement of gastrointestinal symptoms, assessed by sphincter pressure, with a reduction in dysphagia, gastroesophageal emptying and gastroesophageal reflux.

Secondary outcome(s)
The secondary outcome will be adverse effects of the treatments.

Data extraction (selection and coding)
Two reviewers (CS, CBM) will separately and independently screen the titles and abstracts of studies identified from initial searches. A standard screening checklist based on the eligibility criteria above will be used for each study. Studies that do not meet the criteria according to their titles or abstracts will be excluded. Full text versions of the remaining studies, including those that are potentially eligible studies and uncertain, will be retrieved for a second review, by the same two reviewers (CS, CBM) independently to determine the eligibility. Disagreements with regard to study eligibility will be further discussed among reviewers. If consensus cannot be reached, a third reviewer (VC) will make the ultimate decision. For studies with insufficient data to evaluate the eligibility, we will contact the study authors via email to obtain their clarifications. The studies will be excluded if there is still insufficient data after this contact. If more than one publication reports results from the same study population, we will choose the publication with the largest sample size or the one that provides more information. Abstracts published in academic conferences will be evaluated case by case, and we will contact the study authors for details if necessary. The following data will

be collect from each study: general study characteristics (title and authors, year of study, geographical location), methods (study design, participant allocation, measured outcomes reported, covariables), participant's characteristics (age, sex, study inclusion and exclusion criteria), intervention details (dose, period of use) and outcomes (primary and secondary outcomes, time follow-up, number of events). If the study is reported in duplicate, the study published earlier or the one that provided more information will be included.
The same reviewers (CS, CBM) will separately and independently extract data from the eligible studies. Disagreements regarding data extraction between authors will be solved by discussion. If consensus cannot be reached, a third author (VC) will review the study and arbitrate. If data are missing for synthesis or assessment of study quality, we will attempt to contact the study authors via email at least two times. The study will be excluded if there is still insufficient data following this process.

Risk of bias (quality) assessment

Two review authors (CS, CBM) will independently assess for quality. Clinical trials studies will be evaluated according to RoB 2.0: a revised tool for risk of bias in randomized trials (Higgins JPT et al. Revised Cochrane risk of bias tool for randomized trials (RoB 2.0). Seoul Colloquium in October 2016). Observational studies will be assessed for quality using the Risk of Bias In Non-randomized Studies - of Interventions (ROBINS-I) tool (Sterne JA et al. ROBINS-I: a tool for assessing risk of bias in non-randomized studies of interventions. BMJ 2016; 12; ;355: i4919). A table with details of risk of bias in individual studies will be provided.

Strategy for data synthesis

The results will be presented descriptively and the evidence will be incorporated into tabular displays. Variables will be synthetized narratively and summarized using descriptive statistics (frequencies, percentages).
If possible, we will perform meta-analysis, using R program (version 3.2.3) and the meta and metafor package. The relative risk will be computed for the outcomes. Both the fixed effects and random effects models will be applied, using the model appropriate to the methodological heterogeneity between studies. Heterogeneity between studies will be assessed using the Q statistic and the I-squared test. Publication bias across studies will be evaluated using funnel plots and Egger's tests. The overall quality of evidence will be assess using GRADE.

Analysis of subgroups or subsets

If a sufficient number of trials are identified, a subgroup analysis will be performed according to the type of comparator.

Contact details for further information

Cinara Stein
cinarastein@gmail.com

Organisational affiliation of the review

Institute for Education and Research, Hospital Moinhos de Vento, Porto Alegre, Brazil
www.hospitalmoinhosdevento.org.br

Review team members and their organisational affiliations

Miss Cinara Stein. Institute for Education and Research, Hospital Moinhos de Vento, Porto Alegre, Brazil
Miss Celina Borges Migliavaca. Institute for Education and Research, Hospital Moinhos de Vento, Porto Alegre, Brazil
Dr Verônica Colpani. Institute for Education and Research, Hospital Moinhos de Vento, Porto Alegre, Brazil
Dr Luciane Nascimento Cruz. Institute for Education and Research, Hospital Moinhos de Vento, Porto Alegre, Brazil
Dr Maicon Falavigna. Institute for Education and Research, Hospital Moinhos de Vento, Porto Alegre, Brazil

Collaborators

Miss Andreia Diel. Institute for Education and Research, Hospital Moinhos de Vento, Porto Alegre, Brazil

Anticipated or actual start date

11 January 2017

Anticipated completion date

28 April 2017

Funding sources/sponsors

Programa de Apoio ao Desenvolvimento Institucional do Sistema Único de Saúde (PROADI-SUS). Brazilian Ministry of Health

Conflicts of interest

None known

Language

English

Country

Brazil

Stage of review

Review_Ongoing

Subject index terms status

Subject indexing assigned by CRD

Subject index terms

Chagas Disease; Esophageal Achalasia; Humans; Isosorbide; Isosorbide Dinitrate; Nifedipine

Date of registration in PROSPERO

11 January 2017

Date of publication of this version

11 January 2017

Details of any existing review of the same topic by the same authors

Stage of review at time of this submission

The review has not started

Stage	Started	Completed
Preliminary searches	Yes	Yes
Piloting of the study selection process	Yes	No
Formal screening of search results against eligibility criteria	No	No
Data extraction	No	No
Risk of bias (quality) assessment	No	No
Data analysis	No	No

Versions

11 January 2017

PROSPERO
This information has been provided by the named contact for this review. CRD has accepted this information in good faith and registered the review in PROSPERO. CRD bears no responsibility or liability for the content of this registration record, any associated files or external websites.
