# Supplementary material for: Isosorbide and nifedipine for Chagas' megaesophagus: A systematic review and meta-analysis
Source: PLoS Negl Trop Dis. 2018 Sep 28;12(9):e0006836. doi: 10.1371/journal.pntd.0006836 (PMC6179300; doi:10.1371/journal.pntd.0006836)
Supplement: S3 Appendix — (PDF) [file pntd.0006836.s003.pdf]

**SEARCH STRATEGIES:**

**Isosorbide:**

PubMed

1. "Chagas Disease"[Mesh]
2. "chagas disease”
3. "Trypanosoma cruzi"[Mesh]
4. "Trypanosoma cruzi"
5. 1 OR 2 OR 3 OR 4
6. "Isosorbide"[Mesh]
7. Isosorbide
8. 6 OR 7
9. 5 AND 8

Embase

1. 'chagas disease'/exp
2. 'chagas disease'
3. 'trypanosoma cruzi'/exp
4. 'trypanosoma cruzi'
5. 1 OR 2 OR 3 OR 4
6. 'isosorbide'/exp
7. Isosorbide
8. 6 OR 7
9. 5 AND 8

LILACS

Chagas disease AND isosorbide

**Nifedipine:**

PubMed

1. "Chagas Disease"[Mesh]
2. "chagas disease”
3. "Trypanosoma cruzi"[Mesh]
4. "Trypanosoma cruzi"
5. 1 OR 2 OR 3 OR 4
6. "Nifedipine"[Mesh]
7. "Nifedipine"
8. 6 OR 7
9. 5 AND 8

Embase

1. 'chagas disease'/exp
2. 'chagas disease'
3. 'trypanosoma cruzi'/exp
4. 'trypanosoma cruzi'
5. 1 OR 2 OR 3 OR 4
6. 'nifedipine'/exp
7. ‘Nifedipine’
8. 6 OR 7
9. 5 AND 8

LILACS

Chagas disease AND nifedipine
