# Supplementary material for: Isosorbide and nifedipine for Chagas' megaesophagus: A systematic review and meta-analysis
Source: PLoS Negl Trop Dis. 2018 Sep 28;12(9):e0006836. doi: 10.1371/journal.pntd.0006836 (PMC6179300; doi:10.1371/journal.pntd.0006836)
Supplement: S4 Appendix — (PDF) [file pntd.0006836.s004.pdf]

**Methodological quality of before-after studies using the 'Quality Assessment Tool for Before–After (Pre-Post) Studies with No Control Group'**

| Study | 1 | 2 | 3 | 4 | 5 | 6 | 7 | 8 | 9 | 10 | 11 | 12 | *Quality rating* |
|---|---|---|---|---|---|---|---|---|---|---|---|---|---|
| Dantas et al., 1986 | Y | N | Y | NR | NR | Y | Y | N | NR | Y | NA | NA | Fair |
| Dantas et al., 1987 | Y | N | Y | NR | NR | Y | Y | N | NR | Y | NA | NA | Fair |
| Dantas et al., 1988 | Y | N | Y | NR | NR | Y | Y | N | NR | Y | NA | NA | Fair |
| Matsuda et al., 1995 | Y | N | Y | NR | NR | Y | Y | N | NR | Y | NA | NA | Fair |

Abbreviations: Y, yes; N, no; NA, not applicable; NR, not reported.
(1) Objective clearly stated; (2) eligibility criteria described; (3) representative patient population; (4) all eligible participants enrolled in study; (5) sufficient sample size; (6) intervention described; (7) outcome measures specified; (8) outcome assessors blinded; (9) loss to follow-up; (10) statistical analysis of outcome measures before and after intervention; (11) interrupted time-series design; (12) individual data used for group-level effects.

**Methodological quality of crossover trials using the 'RoB 2.0 tool for crossover trials'**

| Study | Randomization | Interventions | Missing outcome data | Outcome Measurement | Reported results | *Overall* |
|---|---|---|---|---|---|---|
| de Oliveira, 1994 | High | Some concerns | Low | High | Low | High |
| Figueiredo, 1992 | Some concerns | Some concerns | Low | High | Low | High |
| Rezende-Filho, 1990 | High | Some concerns | Low | High | Low | High |
| Ferreira-Filho, 1991 | Low | Low | Low | Low | Low | Low |
