# Supplementary material for: Isosorbide and nifedipine for Chagas' megaesophagus: A systematic review and meta-analysis
Source: PLoS Negl Trop Dis. 2018 Sep 28;12(9):e0006836. doi: 10.1371/journal.pntd.0006836 (PMC6179300; doi:10.1371/journal.pntd.0006836)
Supplement: S5 Appendix — (PDF) [file pntd.0006836.s005.pdf]

Lower Esophageal Sphincter Pressure (mmHg)

All patients received isosorbide.

Time (min)	Dantas, 1987			Dantas, 1988 (n=15)		Matsuda, 1995 (n=7)	
	N	Mean	SD	Mean	SD	Mean	SD
**0**	28	19,0	10,58	15,1	6,27	16,1	5,45
**5**	28	12,2	13,87	7,8	7,20	10,0	9,45
**10**	28	6,5	7,94	5,8	6,47	6,9	8,55
**15**	28	4,5	7,46	4,5	7,94	5,5	6,88
**20**	28	5,2	10,37	4,8	7,59	5,6	7,12
**25**	28	5,3	10,11	5,4	10,42	5,6	7,14
**30**	24	5,7	6,42	4,6	6,66	NR	NR
**35**	24	5,3	7,79	4,4	5,89	NR	NR
**40**	18	5,2	6,36	5,1	6,86	NR	NR
**45**	18	6,5	7,08	6,3	6,43	NR	NR
**50**	17	5,6	6,76	5,6	7,40	NR	NR
**55**	17	6,9	10,18	7,1	9,88	NR	NR
**60**	17	8,0	8,74	7,8	8,52	NR	NR

NR: not reported

Esophageal radioativity retention (%)

The intervention is described on the columns.

Time (min)	Rezende-Filho, 1990 (n=18)				Figueiredo, 1992 (n=11)						De Oliveira, 1994 (n=18)			
	Basal		Isosorbide		Basal		Isosorbide		Nifedipine		Basal		Isosorbide	
	Mean	SD	Mean	SD	Median	Range	Median	Range	Median	Range	Mean	SD	Mean	SD
**0**	58,74	NR	36,94	NR	83	5-100	54	5-87	78	7-99	72,51	24,61	51,5	22,58
**5**	40,97	NR	20,22	NR	32	5-86	13	1-68	40	6-90	51,18	24,59	24,63	18,66
**10**	37,54	NR	20,02	NR	28	4-86	10	1-66	34	6-86	NR	NR	NR	NR
**15**	33,95	NR	19,83	NR	29	6-86	9	1-61	37	6-86	NR	NR	NR	NR
**20**	31,52	NR	20,06	NR	29	5-72	9	0-46	34	5-82	NR	NR	NR	NR
**25**	29,66	NR	20,14	NR	NR	NR	NR	NR	NR	NR	NR	NR	NR	NR
**30**	27,06	NR	19,95	NR	NR	NR	NR	NR	NR	NR	NR	NR	NR	NR
**35**	27,04	NR	20,04	NR	NR	NR	NR	NR	NR	NR	NR	NR	NR	NR
**40**	27,19	NR	20,54	NR	NR	NR	NR	NR	NR	NR	NR	NR	NR	NR

NR: not reported
