# Supplementary material for: Isosorbide and nifedipine for Chagas' megaesophagus: A systematic review and meta-analysis
Source: PLoS Negl Trop Dis. 2018 Sep 28;12(9):e0006836. doi: 10.1371/journal.pntd.0006836 (PMC6179300; doi:10.1371/journal.pntd.0006836)
Supplement: S6 Appendix — (PDF) [file pntd.0006836.s006.pdf]

Should we use isosorbide dinitrate in patients with megaesophagus secondary to Chagas disease? Bibliography: Dantas et al, 1987; Dantas et al, 1988; Rezende-Filho, 1990; Ferreira-Filho, 1991; Figueiredo et al, 1992; de Oliveira, 1994; Matsuda, 1995							
Certainty assessment							Summary of findings
No. of participants (studies)	Risk of bias	Inconsistency	Indirectness	Imprecision	Other	Overall certainty of evidence	Impact
**Lower esophageal sphincter pressure (LESP)**							
50 (3 observational studies)	serious [a]	not serious	not serious	serious [b]	none	⨁◯◯◯ VERY LOW	Isosorbide decreased LESP by 10.52mmHg (95%CI −13.57 to −7.47)
**Esophageal emptying (assessed through esophageal retention)**							
47 (3 RCTs)	serious [c]	not serious	not serious	serious [b]	none	⨁⨁◯◯ LOW	Isosorbide decreased esophageal retention by 22.16% (95%CI −29.84 to −14.38)
**Dysphagia**							
23 (1 RCT)	not serious	not serious	not serious	serious [b]	none	⨁⨁⨁◯ MODERATE	Ferreira-Filho 1991: patients reported improvement in frequency and severity of dysphagia when treated with isosorbide, in comparison with placebo and no treatment.

CI: confidence interval **RCT:** randomized clinical trial

Explanations

a. Studies did not report eligibility criteria, loss to follow-up, or sample size estimation; also, there were concerns about blinding of patients and assessors.

b. Small sample size.

c. Problems concerning randomization and blinding.

Should we use nifedipine in patients with megaesophagus secondary to Chagas disease?<br>Bibliography: Dantas et al, 1986; Figueiredo et al, 1992							
Certainty assessment							Summary of findings
No. of participants (studies)	Risk of bias	Inconsistency	Indirectness	Imprecision	Other	Overall certainty of evidence	Impact
**Lower esophageal sphincter pressure (LESP)**							
15<br>(1 observational study)	serious [a]	not serious	not serious	serious [b]	none	⨁◯◯◯<br>VERY LOW	After use of 10mg sublingual nifedipine, LESP decreased up to 60% compared with basal conditions. However, approximately 1/4 of patients showed no changes in LESP.
**Esophageal emptying (assessed through esophageal retention)**							
11<br>(1 RCT)	serious [c]	not serious	not serious	serious [b]	none	⨁⨁◯◯<br>LOW	Use of 20mg sublingual nifedipine did not alter esophageal retention compared with control conditions.

Explanations

a. Study did not report eligibility criteria, loss to follow-up, or sample size estimation; also, there were concerns about blinding of patients and assessors.

b. Small sample size.

c. Problems concerning randomization and blinding.
