# Supplementary figures and images for: Isosorbide and nifedipine for Chagas' megaesophagus: A systematic review and meta-analysis
Source: PLoS Negl Trop Dis. 2018 Sep 28;12(9):e0006836. doi: 10.1371/journal.pntd.0006836 (PMC6179300; doi:10.1371/journal.pntd.0006836)

ISOSORBIDE

HEADACHE

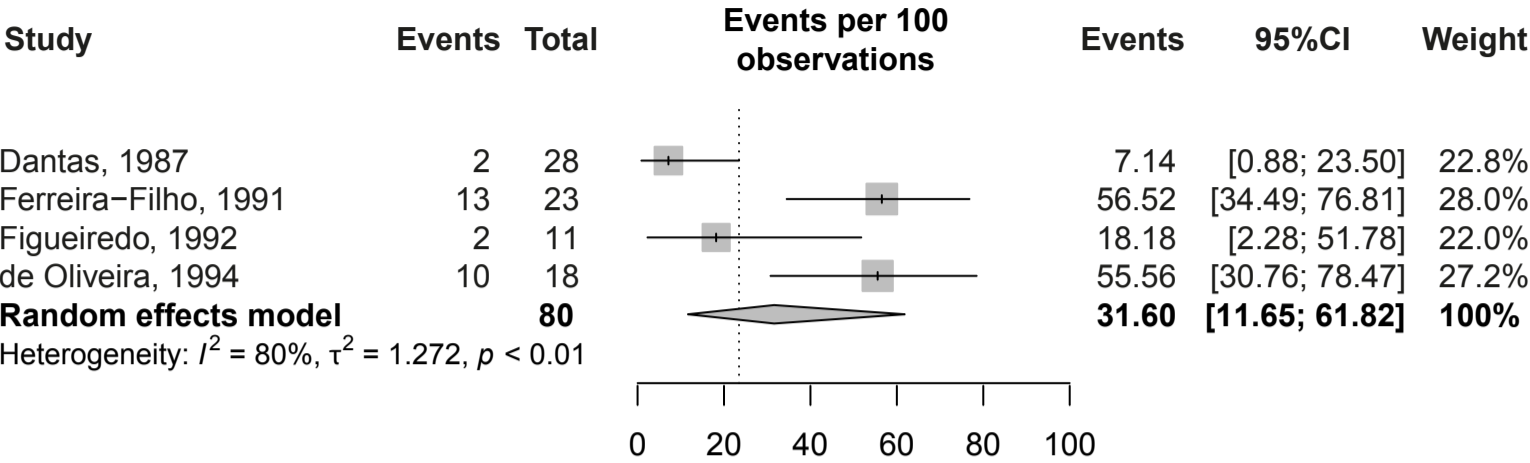

PALPITATION

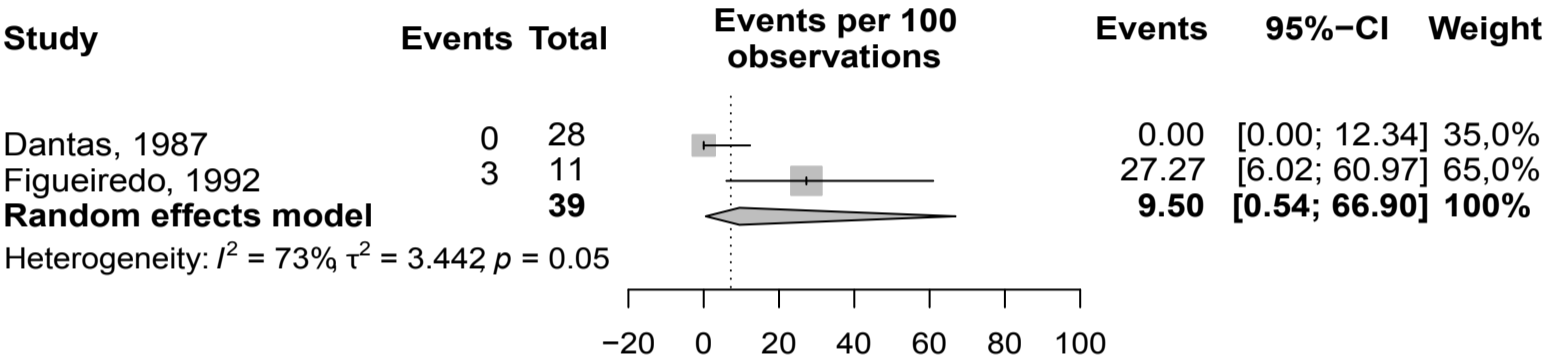

FAINTNESS

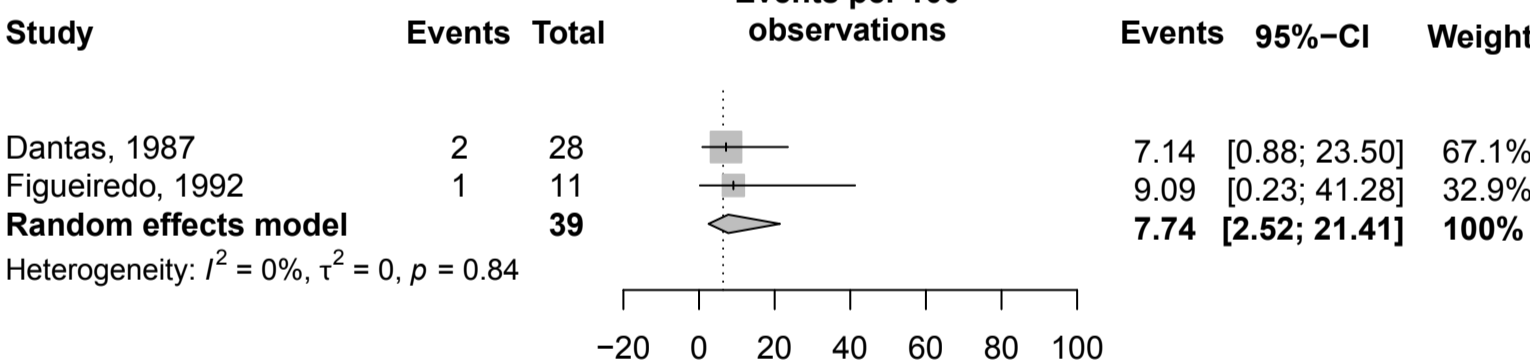

NIFEDIPINE

HEADACHE

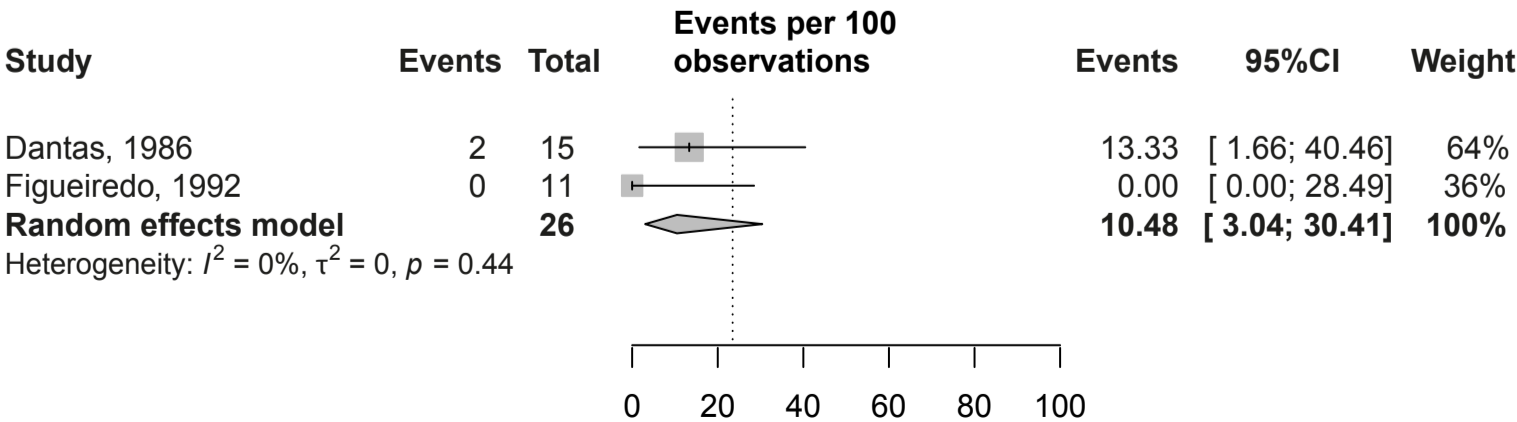

Supplement: S7 Appendix — (PDF) [file pntd.0006836.s007.pdf]
